# Propranolol inhibits myocardial infarction-induced brown adipose tissue D2 activation and maintains a low thyroid hormone state in rats

**DOI:** 10.1590/1414-431X20198491

**Published:** 2019-10-10

**Authors:** F.A.C. Seara, I.G. Araujo, G.E. Império, M.P. Marassi, A.C.M. Silva, A.S. Mecawi, L.C. Reis, E.L. Olivares

**Affiliations:** 1 Departamento de Ciências Fisiológicas, Instituto de Ciências Biológicas e da Saúde, Universidade Federal Rural do Rio de Janeiro, Seropédica, RJ, Brasil; 2 Laboratório de Eletrofisiologia Cardíaca, Instituto de Biofísica Carlos Chagas Filho, Universidade Federal do Rio de Janeiro, Rio de Janeiro, RJ, Brasil; 3 Departmento de Biofísica, Escola Paulista de Medicina, Universidade Federal de São Paulo, São Paulo, SP, Brasil; 4 Programa Multicêntrico de Pós-Graduação em Ciências Fisiológicas, Sociedade Brasileira de Fisiologia, Programa Multicêntrico de Pós-Graduação em Ciências FisiológicasSociedade Brasileira de Fisiologia, Brasil

**Keywords:** Myocardial infarction, Thyroid hormone, Deiodinase, Beta-blocker, Rats

## Abstract

Considering the recognized role of thyroid hormones on the cardiovascular system during health and disease, we hypothesized that type 2 deiodinase (D2) activity, the main activation pathway of thyroxine (T4)-to-triiodothyronine (T3), could be an important site to modulate thyroid hormone status, which would then constitute a possible target for β-adrenergic blocking agents in a myocardial infarction (MI) model induced by left coronary occlusion in rats. Despite a sustained and dramatic fall in serum T4 concentrations (60–70%), the serum T3 concentration fell only transiently in the first week post-infarction (53%) and returned to control levels at 8 and 12 weeks after surgery compared to the Sham group (P<0.05). Brown adipose tissue (BAT) D2 activity (fmol T4·min^-1^·mg ptn^-1^) was significantly increased by approximately 77% in the 8th week and approximately 100% in the 12th week in the MI group compared to that of the Sham group (P<0.05). Beta-blocker treatment (0.5 g/L propranolol given in the drinking water) maintained a low T3 state in MI animals, dampening both BAT D2 activity (44% reduction) and serum T3 (66% reduction in serum T3) compared to that of the non-treated MI group 12 weeks after surgery (P<0.05). Propranolol improved cardiac function (assessed by echocardiogram) in the MI group compared to the non-treated MI group by 40 and 57%, 1 and 12 weeks after treatment, respectively (P<0.05). Our data suggested that the beta-adrenergic pathway may contribute to BAT D2 hyperactivity and T3 normalization after MI in rats. Propranolol treatment maintained low T3 state and improved cardiac function additionally.

## Introduction

Ischemic heart disease remains the leading cause of death worldwide (1). Overall prognosis has been aggravated by limitations of available therapies and poor cardiac healing capacity. These limitations have fueled the search for alternative therapeutic strategies with focus in non-classical systems implicated in heart failure (HF) pathophysiology. One of these promising “non-classical” systems is the thyroid system (2). Thyroid hormones (TH) play important roles in cardiovascular homeostasis (3). In most cases, after myocardial infarction (MI), serum triiodothyronine (T3) can be decreased, with no changes on thyroxin (T4) levels, a condition known as low T3 syndrome (4).

Most symptoms resemble a hypothyroid state, i.e., chronic fatigue, mood disorders, swelling, skin disorders, dyslipidemia, and decreased metabolic rate. Cardiovascular repercussions include decreased heart rate and cardiac contractility, as well as mild increases in peripheral vascular resistance. However, thyroid hormone replacement therapy has shown some benefits in patients with ischemic heart disease and recent evidence has suggested that low T3 status could be a necessary allostatic response to reduce the metabolic rate of ischemic heart. Indeed, either systemic or cardiac hypothyroidism elicited by HF can be partially associated with the induction of ectopic cardiac type 3 deiodinase (D3) activity, the main inactivating pathway of TH (5). These features confirm the pathophysiology of “consumptive hypothyroidism,” which has been previously described in patients with large D3-expressing tumors (6), and do not support the “low T3-syndrome” model proposed in patients with HF (7,8). Even so, despite the persistent decrease of circulating T4 levels, T3 levels can return to basal levels in late phases of MI, i.e., 8 and 12 weeks post-surgery (9).

Although high expression levels of ectopic cardiac D3 activity have been initially proposed to explain hypothyroidism following MI, the mechanism whereby T3 can be normalized after MI remains unclear. Because type-1 deiodinase (D1) activity remains low throughout 12 weeks of MI, the type 2 deiodinase (D2) pathway has become the potential mechanism underlying, at least in part, the progressive re-establishment of serum T3 after MI (9). Both D1 and D2 can convert T4 into T3 and, although D2 is crucial for local T3 production, it can also affect circulating T3 levels (10–13). In rats, either Dio2 mRNA expression or D2 activity have been mostly observed in the anterior pituitary gland, cerebral cortex (14), and brown adipose tissue (BAT) (15), although it has also been detected in thyroid and skin (16,17). Thus, unsurprisingly, BAT has been widely investigated as a potential contributor to maintain circulating T3 within physiological ranges (18–21). Together, these physiological properties have fueled studies to better understand the roles of BAT on thyroid homeostasis over cardiovascular diseases and to seek new pharmacological approaches for HF patients.

It has been demonstrated that D2 activity can be modulated by several factors, including sympathetic stimulation (22). Because MI and HF can induce sympathetic overactivation (23) and hypothyroidism (9), we hypothesized that BAT D2 activity could contribute to the reestablishment of circulating T3 levels during the progression of HF following MI. Although the role of sympathetic overactivity in the pathophysiological progression of HF, as well as the therapeutic benefits of beta-adrenergic blockers have been widely demonstrated, there has been little evidence on the repercussion of these drugs on BAT D2 activity and TH economy, particularly during HF. Therefore, we aimed to investigate whether: i) the D2 pathway is overactivated following MI and ii) treatment with propranolol (the first successful beta-blocker developed) can influence the thyroid hormone economy and somehow partly explain its beneficial effects in a MI model.

## Material and Methods

### Animals

Male Wistar rats (200–250 g) were maintained in cages (4 per cage) in a room under controlled temperature (24±2°C) and lighting (lights on from 6:00 to 18:00 h), with free access to food and water. Animal handling and experimental procedures were performed according to the Guide for the Care and Use of Laboratory Animals published by the US National Institutes of Health (NIH Publication No. 85–23, revised 1996) and the Institutional Committee of Ethics and Animal Welfare (CEAW number: 23083.004836/20120-58).

### Experimental myocardial infarction

MI was induced following the procedure previously described and modified by our group (9). After anesthesia (Isoflurane, Biochimico^®^, Brazil), a skin incision was performed at the left parasternal level, followed by dissection of the pectoralis major and minor muscles. The incision was made between the 4 or 5th left intercostal spaces, through which the heart was externalized. Left coronary artery was located and ligated with a 6-0 silk suture, as close as possible to its origin on the aorta. The heart was then quickly placed in its original anatomical position. Sham-operated group (Sham) was subjected to the same surgical procedure as the infarcted group but without left anterior coronary artery occlusion. Prophylactic doses of veterinary antibiotic (0.2 mL, *im*, Pentabiótico Veterinário Pequeno Porte^®^, Fort Dodge, Brazil) and analgesic flunixin meglumine (2.5 mg/kg, *im*, Banamine^®^, Schering-Plough, Brazil) were given.

### Heart function assessment and pathology

Electrocardiogram (ECG) was registered one day after MI and echocardiogram (ECHO) evaluations were performed 1, 6, and 12 weeks after surgery to assess cardiac function. Echocardiograph color-system (Megas/Esaote, Italy) equipped with a 10 MHz electronic-phased-array transducer was used. Images were obtained from the left parasternal and apical windows. Short-axis 2-dimensional views of the left ventricle (LV) were taken at the level of papillary muscles to obtain the M-mode recordings. Systolic function was expressed by the ejection fraction (EF, %), calculated by the Simpson's method, after the LV volume calculation: systolic and diastolic LV long axis were measured on long-axis view, and systolic and diastolic LV short axis, traced at the level of papillary muscle, were measured on transversal view.

Pathological analysis was performed as previously described (9). Heart weight (HW), lung weight (LW), and liver weight (LiW) relative to body weight (BW) were calculated. Hearts were perfused with 4% paraformaldehyde in phosphate buffer. LV samples were sliced from apex to base (1–2 mm), and the slices were labeled as A (at the apex), B, C, and D. Hematoxylin-eosin and Picrosirius staining were performed in representative sections obtained from slice C, described as the most representative of the total infarcted length using an Axiovert 100 microscope (Zeiss Inc., Germany). Sections stained with Picrosirius were recorded with a digital camera and stored for later analysis. All digital files were analyzed with ImageJ software (version 1.27 z, National Institutes of Health, USA). The percentage of the infarcted endocardium and epicardium was calculated, and the average infarct size percentage was estimated.

### Radioimmunoassay (RIA) for T3 and T4

Serum T3 and T4 were determined by specific Coated-Tube RIA kits (T3: DLS–3100 Active TX, USA; T4: DLS–3200 Active TX, USA). All procedures were performed following the recommendations of the respective kit.

### D2 activity

Pituitary gland or BAT (40 mg) were homogenized with 0.5 mL (pituitary glands) or 1 mL (BAT) of 0.1 M sodium phosphate buffer containing 1 mM EDTA, 0.25 M sucrose, and 10 mM dithiothreitol, pH 6.9. Each homogenate sample (100 μL containing 150 μg protein for BAT samples and 50 μg for pituitary samples) was incubated for 3 h at 37°C with 1 nM [^125^I] T4 (Perkin Elmer Life and Analytical Sciences, USA), 20 mM dithiothreitol, and 1 mM propylthiouracil (PTU) in 100 mM potassium phosphate buffer, pH 6.9, containing 1 mM EDTA.

Total reaction volume was 300 μL. The reaction was stopped at 4°C in ice-bath followed by addition of 200 μL fetal bovine serum (Cultilab, BR) and 100 μL trichloroacetic acid (50%, v/v), and vigorous agitation. The samples were centrifuged at 8000 *g* for 3 min at 4°C, and an aliquot of the supernatant (360 μL) was collected to measure ^125^I liberated during deiodination reaction. Protein concentration was measured by the Bradford method after incubation of homogenates with NaOH (2.5 N). Enzyme activity is reported as femtomoles of T4 deiodinated per min per mg of protein.

### Experimental design

#### Phase I: Heart function, T3 and T4 serum concentrations, and D2 activity in male rats subjected to experimental MI

Wistar rats underwent MI (MI group, n=20) or sham operation (Sham group, n=18) and serial blood sample collections (1, 8, and 12 weeks after surgery) through the jugular vein to measure serum T3 and T4 concentrations. ECHO exams were performed on the first and twelfth week post-surgery, immediately before blood collection. At the end of 1, 8, and 12 weeks after the surgical procedure, the animals were randomly euthanized by decapitation after sedation (n=4-10/time/group) and the tissues (BAT and pituitary gland) were collected for D2 activity measurement.

#### Phase II: Influence of chronic *β*-adrenergic blockade on heart function, T3 and T4 serum concentrations, and D2 activity in male rats subjected to experimental MI

Wistar rats underwent MI (n=32) or Sham (n=28) procedures and were randomly assigned to four groups: rats treated daily with propranolol hydrochloride (Sigma Aldrich), a β-adrenergic antagonist in drinking water (0.50 g/L): Sham+Prop (n=14) and MI+Prop (n=16) or vehicle: Sham+water (n=14) and MI+water (n=16), during 1 (n=7–8/group) or 12 (n=7–8/group) weeks after surgery. At the end of each protocol, all groups underwent ECHO analyses and were euthanized the next day. Blood and BAT samples were collected for T4 and T3 serum measurement by radioimmunoassay and D2 activity assessments, respectively.

### Statistical analyses

Data are reported as means±SE. Statistical analyses were performed by unpaired Student’s *t*-test or one-way ANOVA, followed by Bonferroni multiple comparison post-test, using GraphPad Prism^®^ 4 (GraphPad Software, Inc., USA). Pearson's product moment correlation was used to assess the relationship between the D2 activity and the T3/T4 ratio in each period, and a Student’s *t*-test for paired data was used to assess the significant differences. Differences were considered statistically significant for P<0.05.

## Results

### Phase I

#### ECG and heart function assessment

**Figure 1.**
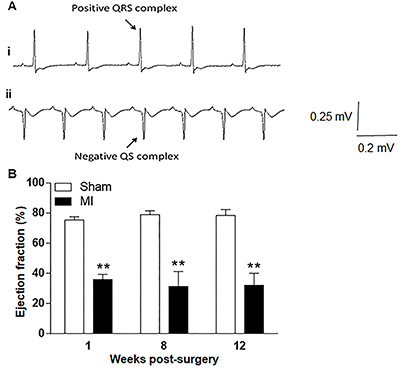
Characterization of myocardial infarction (MI)-induced heart failure model. Representative ECG (**A**) from one Sham animal (i) and one MI animal (ii). The negative QS complex characteristic of transmural MI is highlighted in ii. Progression of ejection fraction (EF, %) of MI and Sham-operated groups 1, 8, and 12 weeks after surgery are demonstrated in **B**. Data are reported as means±SE. **P<0.01 *vs* respective Sham group at the same week (one-way ANOVA followed by Bonferroni multiple comparison post-test).

ECG and ECHO validated the efficiency of the MI model. As expected, MI was characterized by rightward deviation of the frontal QRS axis (ÂQRS), Q wave was observed in L1, and QRS index (I-QRS) decreased (Figure 1A). Animals that did not show ECG signs of MI (two animals) were excluded from the study. ECHO (Figure 1B) showed sustained reduction (approximately 40–50%) of LV EF in all observed periods in infarcted animals compared to the Sham group.

#### RIA for T3 and T4

**Figure 2.**
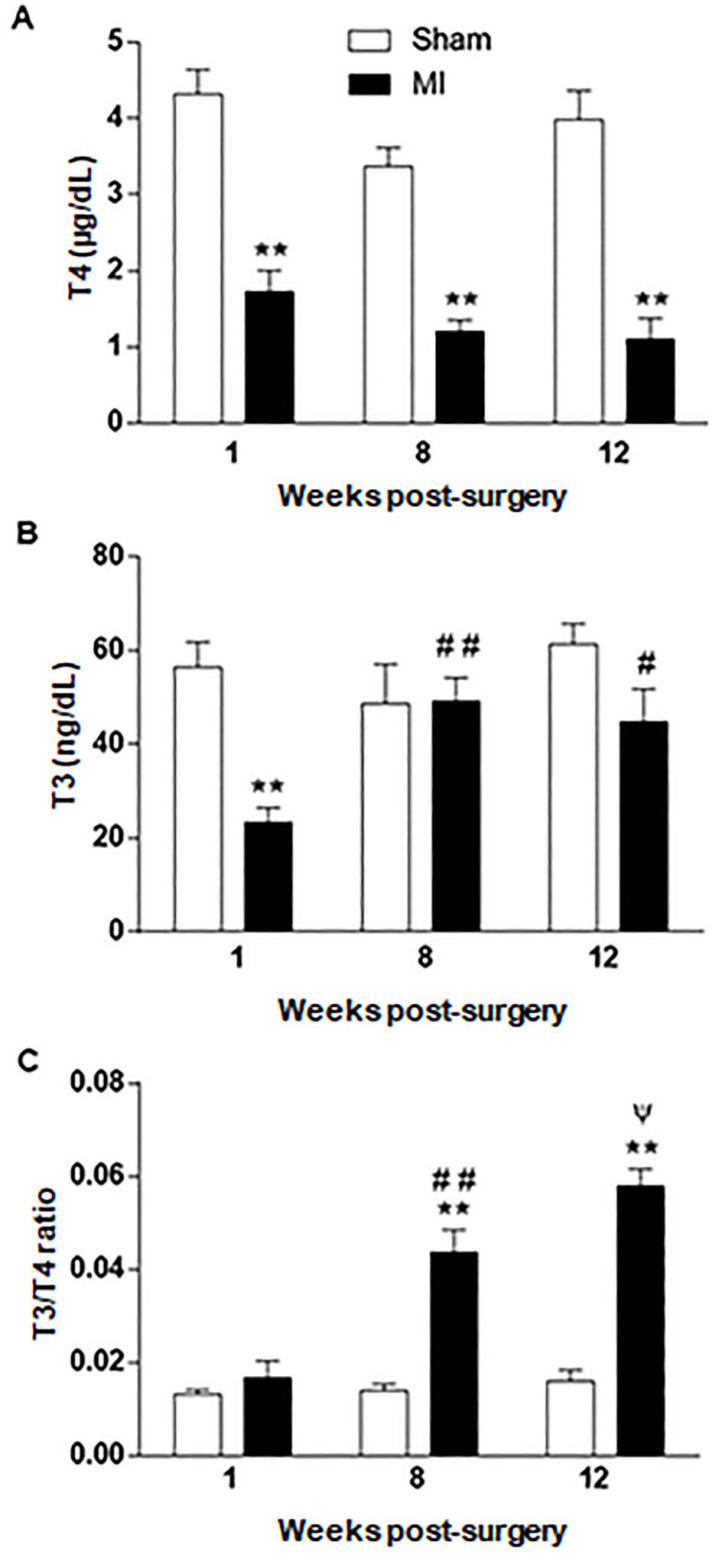
Circulating thyroid hormone concentration of Sham and infarcted (MI) groups. Circulating T4 (**A**) and T3 (**B**) concentration of Sham-operated and MI groups at weeks 1, 8, and 12 post-surgery was measured by radioimmunoassay. T3/T4 ratio is reported in **C**. Data are reported as means±SE. **P<0.01 *vs* respective control group at the same week; ^#^P<0.05 and ^##^P<0.01 *vs* MI group one week after surgery; ^ψ^P<0.05 *vs* MI group 8 weeks after surgery (one-way ANOVA followed by Bonferroni multiple comparison post-test).

Despite sustained decrease of serum T4 concentrations (approximately 60-70%) of MI compared to the Sham group since the first week post-surgery (Figure 2A), serum T3 concentration fell only transiently in the first week (P<0.01) and returned to control levels at 8 (P>0.05) and 12 weeks (P>0.05) after surgery (Figure 2B). Additionally, T3/T4 ratio was increased in MI rats *vs* Sham group at 8 and 12 weeks after surgery (Figure 2C). T3/T4 ratio was also higher in the 8th and 12th weeks compared to values observed in the MI group in the first week post-infarction (P<0.01).

#### D2 activity

**Figure 3.**
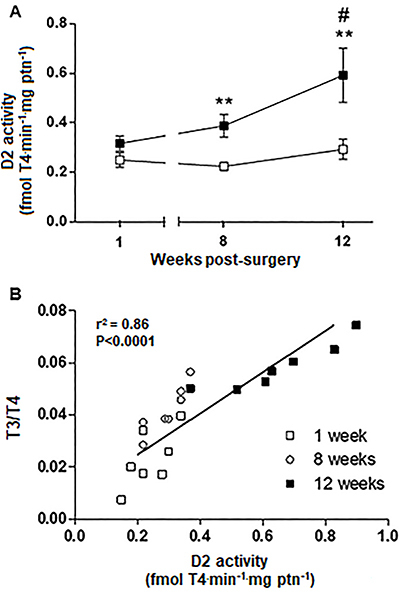
Temporal effects of myocardial infarction (MI; black squares) on type 2 deiodinase (D2) activity. Progression of D2 activity in brown adipose tissue (BAT) (**A**) and correlation between the triiodothyronine/thyroxine (T3/T4) ratio and D2 activity in BAT (**B**) at 1, 8, and 12 weeks of sham-operated (Sham) or infarcted rats over the 12 weeks of the experimental protocol. Data are reported as means±SE. **P<0.01 *vs* respective control group; ^#^P<0.05 *vs* MI group in the first week. **B**, Point of intersection between the D2 activity in BAT and the T3/T4 ratio for each animal studied.

BAT D2 activity (Figure 3A) increased at 8 weeks (P<0.01) and 12 weeks (P<0.05) after MI *vs* Sham group, with no differences in the first week (P>0.05). D2 activity progressively increased only in infarcted rats (Figure 3B), whereas no changes in pituitary D2 activity were noted (data not shown). A strong and positive correlation was found between the T3/T4 ratio and BAT D2 activity in the MI group (P<0.0001) over the course of the 12 weeks after the permanent coronary occlusion (Figure 3B).

### Phase II

#### Heart function assessment and pathology

**Figure 4.**
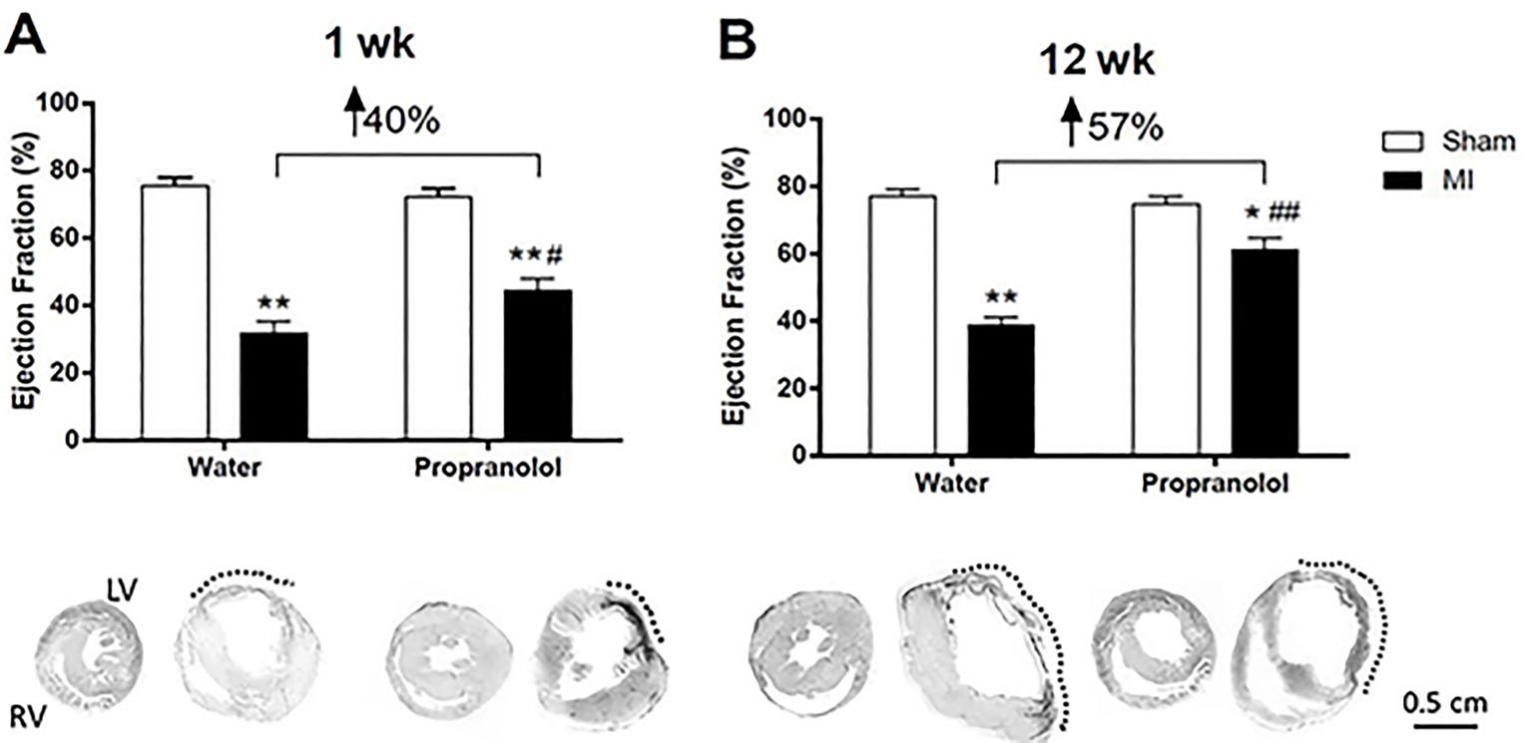
Effect of propranolol treatment on heart function and the infarct area. Ejection fraction (EF) was assessed by echocardiography in Sham-operated and myocardial infarction (MI) groups treated with water or β-blocker propranolol at weeks 1 (**A**) and 12 (**B**). Picrosirius staining performed in representative heart sections obtained from all groups (lower part). The infarct area (scar tissue) in LV is delimited by broken lines. As shown in Table 1, in these representative hearts, propranolol treatment reduced the infarct size at the first week post-MI (31 *vs* 40%), but not 12 weeks post-MI (45 *vs* 48%) compared to MI treated with vehicle, respectively. Note the thickening of the scar in propranolol-treated infarcted rats, especially at week 1. Representative histopathological slides from the hearts are shown in the same sequence as panels **A** and **B**. LV: left ventricle; RV: right ventricle. Scale bar=0.5 cm. Data are reported as means±SE. *P<0.05 and **P<0.01 *vs* respective control group at the same week. ^#^P<0.05 and ^##^P<0.01 *vs* MI+Water group (one-way ANOVA followed by Bonferroni multiple comparison post-test).

Propranolol prevented MI-induced tachycardia at 8 and 12 weeks post-surgery (P<0.05), suggesting that cardiac adrenergic stimulation was blunted. ECHO analysis (Figure 4) demonstrated sustained reduction of LV EF (approximately 40–50% decrease) in both periods of observation in MI compared to the Sham group. Cardiac function was improved by propranolol, including LV EF one and 12 weeks post-MI (Figure 4). However, this improvement was greater at 12 weeks (57%) than 1 week (40%) post-MI.

Pathology data (Table 1) showed that HW was significantly increased in the MI+Water group compared to the Sham+Water group at 1 and 12 weeks after infarction surgery (P<0.05), suggesting the occurrence of cardiac hypertrophy. Conversely, MI+Propranolol exhibited no increase of HW in the first week and a slight, but significant increase 12 weeks after infarction sugery compared to control (P<0.05). Increased LW was also evident in MI+Water at both time periods, consistent with congestive failure, which was less apparent in MI+Propranolol-treated animals. Again, propranolol treatment was more effective in the first week compared to 12 weeks post-MI. LiW did not change throughout the experimental protocol in all groups.

**Table 1.** Body weight, heart, lung, and liver weights, and infarct size at 1 and 12 weeks.

	Body weight (g)	Relative HW (mg/g)	sRelative LW (mg/g)	Relative LiW (mg/g)	Infarct size (%)
Parameters at week 1					
Sham+Water	260.9±3.18^a^	3.4±0.14^a^	5.2±0.18^a^	35.9±1.06^a^	
MI+Water	264.4±3.33^a^	5.7±0.23^b^	7.1±0.17^b^	37.7±1.32^a^	44.38±1.47^a^
Sham+Propranolol	261.6±2.73^a^	3.6±0.15^a^	5.4±0.14^a^	35.6±1.59^a^	
MI+Propranolol	266.4±3.53^a^	4.3±0.15^c^	6.1±0.10^c^	39.4±1.62^a^	35.88±1.82^b^
Parameters at week 12					
Sham+Water	450.3±3.91^a^	4.0±0.09^a^	5.8±0.16^a^	37.4±1.16^a^	
MI+Water	467.3±3.87^a^	6.2±0.30^b^	8.4±0.13^b^	39.3±1.30^a^	50.86±2.20^a^
Sham+Propranolol	454.4±4.36^a^	4.2±0.21^a^	5.5±0.16^a^	38.2±1.37^a^	
MI+Propranolol	461.4±3.35^a^	5.5±0.15^b^	7.4±0.20^c^	41.7±1.52^a^	48.38±1.82^a^

MI: myocardial infarction; HW: heart weight; LW: lung weight; LiW: liver weight. Data are reported as means±SE. Different letters indicate significantly different data (P<0.05, one-way ANOVA followed by Bonferroni multiple comparison post-test).

Propranolol reduced the infarct size at the first week post-MI (P<0.05) but not 12 weeks post-MI (P>0.05) as observed in MI+Propranolol *vs* MI+Water at both times (Table 1 and Figure 4, lower part). These combined data suggest that cardiac hypertrophy, congestive HF and infarct size do not explain the improvement in cardiac function observed in the MI animals treated with propranolol at 12 weeks. Therefore, other factors should explain the improvement in cardiac function observed in propranolol-treated infarcted rats, particularly in the late phase of cardiac failure.

#### RIA for T3

**Figure 5.**
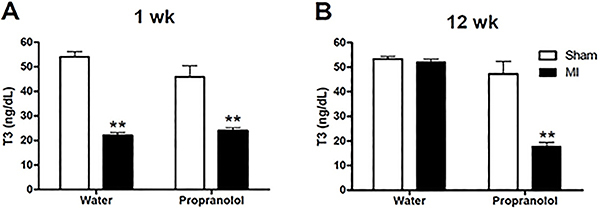
Effect of propranolol on circulating triiodothyronine (T3) levels. Circulating T3 concentration of Sham-operated and myocardial infarction (MI) groups treated with water or propranolol at weeks 1 (**A**) and 12 (**B**) was assessed by radioimmunoassay. Data are reported as means±SE. **P<0.01 *vs* respective control group at the same week (one-way ANOVA followed by Bonferroni multiple comparison post-test).

Serum T3 levels observed in MI-animals in this Phase (Figure 5) reproduced the results shown in Phase I (Figure 2). T3 concentrations fell transiently in the first week post-infarction (approximately 67% decrease) and returned to control levels at 12 weeks. Propranolol did not alter the decrease in T3 levels at 1 week post-MI (approximately 57% decrease), but it was otherwise effective preventing plasma T3 restoration observed in MI-treated rats 12 weeks post-MI (Figure 5). The MI group treated with the β-blocker had values resembling hypothyroid status as observed in non-treated MI group at 1-week post-MI (approximately 66% decrease in T3 levels). Altogether, these data suggested that propranolol treatment for 12 weeks prevented the reestablishment of euthyroid status normally observed in response to MI.

#### D2 activity

**Figure 6.**
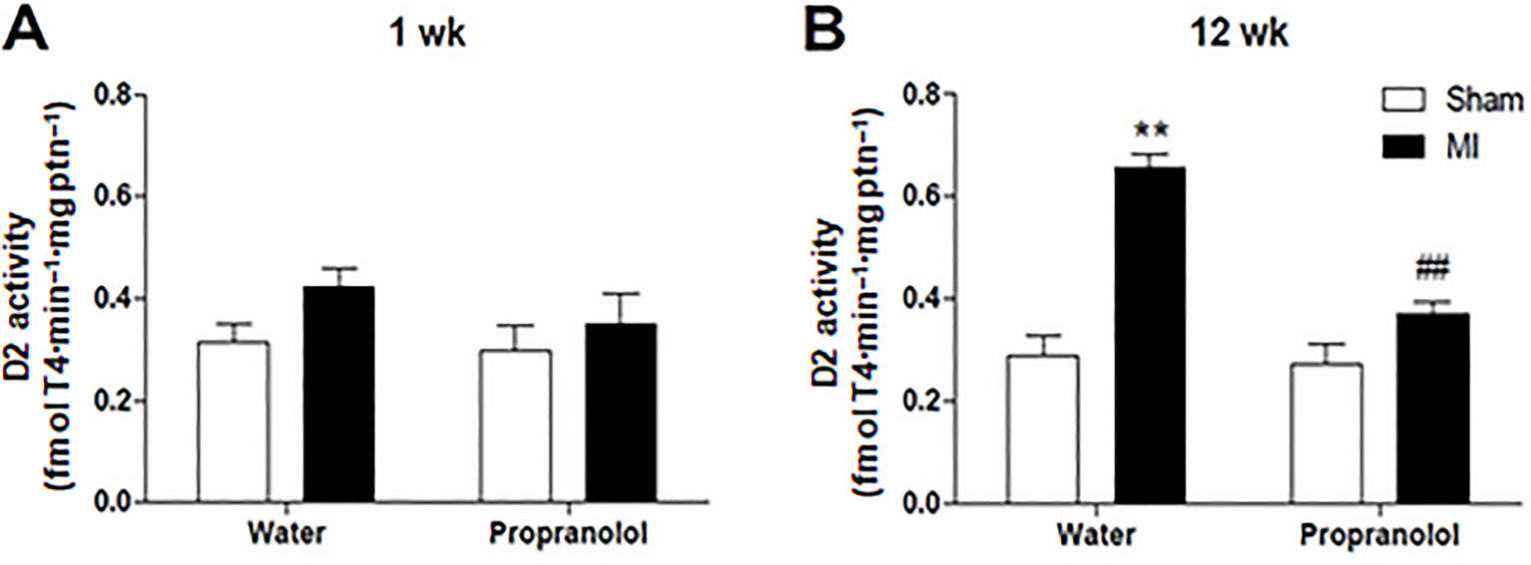
Effect of propranolol on brown adipose tissue (BAT) type 2-deiodinase activity (D2) post-myocardial infarction (MI). BAT D2 activity was assessed in Sham-operated and MI groups treated with water or propranolol at weeks 1 (**A**) and 12 (**B**). Data are reported as means±SE. **P<0.01 *vs* respective control group; ^##^P<0.01 *vs* MI group in the first week (one-way ANOVA followed by Bonferroni multiple comparison post-test).

As expected, BAT D2 activity increased in MI+Water compared to the Sham+Water group at 12 weeks (Figure 6B, P<0.01). MI+Propranolol showed no difference compared to Sham+Propranolol at 12 weeks after the permanent coronary occlusion (Figure 6B, P>0.05). No difference was observed among the groups at 1-week post-surgery (Figure 6A, P>0.05).

## Discussion

This study provides experimental evidence on the role played by BAT D2 activity over TH economy during the progression of HF following MI. BAT D2 activity progressively increased after MI, and it was followed by reestablishment of circulating T3 levels, independently of T4 levels. The increased T4 to T3 conversion could correspond to an important mechanism responsible for serum T3 normalization observed at the late phase of MI, for previous evidence has suggested that D2 might also be important for serum T3 supply, besides its crucial role in intracellular T3 levels (10,14,24). These events were prevented by treatment with the β-blocker propranolol, corroborating the hypothesis that sympathetic activity plays a crucial role to increase BAT D2 activity and, thereby, reestablish circulating T3 levels. Furthermore, the maintenance of low T3 levels was followed by improvement of cardiac function.

As expected, cardiac function was significantly impaired early after coronary ligation, as evidenced by decreased EF at week 1, and this condition persisted towards the following weeks. Circulating TH levels were found decreased after 1 week in MI group, corroborating previous evidence on the development of hypothyroidism after ischemic heart disease. However, while T4 levels remained decreased during the following weeks, circulating T3 levels progressively increased to reach control levels at week 8, resulting in increased T3/T4 ratio. These findings indicated that T3 levels were normalized. In normal conditions, 50% of the intracellular T3 comes from deiodination processes induced by D2 (25), and a drastic reduction of circulating T4 levels would decrease intracellular T3 levels, consequently elevating BAT D2 activity (24). Despite reductions of circulating T3 and T4 levels (50 and 90%, respectively), normal cardiac T3 levels have been demonstrated in a model of iodine deficiency in rats (26). Accordingly, local conversion of TH can be increased by low circulating T4 levels, and BAT can increase as an alternative source for circulating T3 (27).

In keeping with this, BAT D2 activity progressively increased after MI, and it was positively correlated with the T3/T4 ratio. The progressive increase in T4 to T3 conversion was expected, in as much as increased D2 activity can be associated with the longer half-life of D2 protein and increased Dio2 gene transcription (28). However, although circulating T3 levels were normalized, cardiac function of infarcted rats was not improved, corroborating the evidence that infarcted hearts might be less responsive to TH (29). Any difference in pituitary D2 activity was noted in the eighth and twelfth weeks after MI, despite the hypothyroxinemic status of infarcted rats (data not shown). Pituitary D2 can be affected by variations of T4 serum concentrations (24), which may be important for maintenance of D2 homeostasis (mainly in the cerebral cortex) and, thereby, intracellular T3 concentrations (30). Additionally, TSH secretion can be finely modulated by intra-pituitary D2-induced T4 to T3 conversion and by serum T3 levels (24,31). Some reports have also shown that large bolus of T4 can only inhibit TSH secretion if pituitary D2 activity is fully active, strongly supporting the concept of a crucial role of D2 in the control of TSH secretion in normal rats (32). The roles of other Dio2-positive tissues, such as skeletal muscle and heart on T3 restoration after MI remain elusive (33).

D2 activity can be modulated by several factors, such as hypothyroidism, α- and β-adrenergic activation, and bile acids (11 [Bibr B12],^34^). Since MI can be followed by autonomic imbalance, increased BAT D2 activity could be secondary to sympathetic nervous system overactivity (23). Both α- and β-adrenergic receptors can be expressed by brown adipocytes (35), and the enhanced sympathetic activity can effectively modulate BAT metabolism, particularly D2 activity, as observed in cold-stress exposure (36). Because sympathetic overactivity can persist many weeks after permanent coronary occlusion (23), we hypothesized that in addition to hypothyroidism stimuli *per se*, increased BAT D2 activity could also be induced by the higher sympathetic activity towards BAT. Indeed, early stage HF-induced hypothyroidism was not significantly affected by propranolol, though serum T3 normalization in the late stage of cardiac disease was prevented, corroborating the hypothesis that T4 to T3 deiodination was decreased by such pharmacological intervention. These data suggest that the β-adrenergic pathway plays a crucial role on BAT D2 hyperactivity and serum T3 normalization at late stages of HF pathophysiological progression. Future studies employing surgical or pharmacological sympathectomy will be needed to further address this hypothesis.

Another potential mechanism to explain serum T3 normalization lies in D3 activity. If high cardiac D3 activity is associated with low serum thyroid hormones in the early phase of HF (37), T3 normalization observed in the late phase could be secondary to D3 inactivation. Additionally, we characterized and confirmed the consumptive hypothyroidism status, which has been previously described in patients with large D3-expressing tumors (6), as thyroxine injected early after MI could restore euthyroid status only 6 weeks after the beginning of the hormonal treatment. Indeed, there has been no evidence of persistent D3 induction in models of MI in rats (2). Future studies assessing a time course of D3 activity in this model are needed. In addition, D1 activity could be affected by propranolol, since T4 to T3 conversion is also attributed to D1 activity. However, persistent low D1-activity 4 and 12 weeks after MI has already been described (9). Therefore, the possible effect of propranolol on D1 activity is not reasonable, as D1 activity is already down-regulated in our model. Furthermore, it is also not rational to postulate a propranolol effect (block) on D3 activity, since D3 is the main physiological inactivator of TH (31). At the same time, as far as we know, there is no description of beta-blockers on D3 activity in the literature so far.

Beta-adrenergic blockers therapy has long been demonstrated to improve survival in patients with HF (38). Cardiac EF remained significantly decreased at early stages post-MI despite propranolol treatment. Surprisingly, the most striking improvement of cardiac function observed in the MI group treated with propranolol occurred in the late stage (12 weeks post-MI) and it was not correlated with improvement of cardiac remodeling or blood congestion. Since MI was associated with hypothyroidism only in propranolol-treated rats at the end of 12 weeks, these data suggest that beta-blockers maintain a hypothyroid state, guaranteeing additional improvement in cardiac function. The relationship between hypothyroidism and cardiac function has been previously described. Ueta et al. (39) showed that a 10-day sympathetic overdrive worsened the phenotype of restrictive cardiomyopathy in D3KO mice (HtzD3KO) and lends itself as a model of cardiac-specific Dio3 inactivation and local hyperthyroidism. Consequently, LV dysfunction was further impaired, particularly diastolic properties, resulting in congestive HF and a higher mortality rate. In sharp contrast, wild-type siblings could recruit myocardial D3, which resulted in adaptive cardiac remodeling and better cardiac function compared to D3KO mice. Furthermore, we recently demonstrated that hyperthyroidism furthered LV stunning while hypothyroidism was mostly followed by improved post-ischemic recovery of LV hemodynamic properties and decreased infarct size (40). These findings are consistent with our present work, which suggested that cardiac hypothyroidism is an adaptive condition important for guaranteeing an allostatic condition in HF.

### Conclusions

In conclusion, our results suggest that the normalization of serum T3 concentrations after MI in rats can be induced by a progressive increase of D2 activity in BAT, which was potentially induced by the beta-adrenergic pathway post-MI in rats. When this pathway was blocked by propranolol, the low thyroid hormone state persisted, and cardiac function was improved additionally, suggesting a new beneficial effect of beta-blockers in a HF model.
